# Effects of different clones and inducing time on agarwood quality in grafted Qi-Nan *Aquilaria sinensis* (Lour.) spreng

**DOI:** 10.1371/journal.pone.0327514

**Published:** 2025-07-03

**Authors:** Ming Li, Zinong Yang, Zhou Hong, Daping Xu, ZhiHui Li, Shengjie Wang

**Affiliations:** The Key Laboratory of National Forestry and Grassland Administration for Tropical Forestry Research, Research Institute of Tropical Forestry, Chinese Academy of Forestry, Longdong, Guangzhou, China; Central University of Punjab, INDIA

## Abstract

Agarwood is the resinous part of the injured tree of the *Aquilaria* species of the *Thymelaeceae* family, Qi-Nan is a type of agarwood that forms easily and benefits from a shortened inducing time, noted for features like its substantial resin, (2-phenylethyl) chromone and 2-[2-(4-methoxyphenyl) ethyl] chromone content. Although the content of agarotetrol in Qi-Nan is very low, it is still an important indicator for the agarwood quality. A validated method was developed to precisely quantify both agarotetrol and two aforementioned chromones, as well as ethanal extracts, within Qi-Nan to explore how the inducing time (half a year, one year, and two years) and different clones (YYZ, AS, RH, XGY) affect agarwood quality. Different clones and inducing time both have significant effects on agarwood quality. The agarwood quality of Qi-Nan does not necessarily improve with a longer inducing time. YYZ, AS, and RH clones reached to the best agarwood quality after two years of induction. The ethanol extracts content was among 46.82 ~ 48.59%, two chromones were among 10.57% ~ 14.05% after two years of induction. However, for XGY clone, the quality of agarwood after one year of induction was better than that after two years, which reached to the highest ethanol extracts (51.49%) and two chromones content reached to 12.43% and 12.91% after one year of induction. Therefore, for different clones of Qi-Nan, harvesting should be conducted at different time of induction for agarwood formations, which has theoretical implications for the cultivation and industrial development of cultivated Qi-Nan agarwood.

## 1. Introduction

Agarwood is a fragrant natural product rich in resin, aromatic dark resinous heartwood of the *Aquilaria* tree species, created through a self-defense mechanism in response to various types of stress, whether physical, chemical, or biological [1]. Contemporary pharmacological research has demonstrated that agarwood possesses significant effects on the nervous system and is used to effectively treat anxiety and depression with low toxicity and strong safety profiles in clinical settings [2]. In addition, agarwood has shown significant biological activities including gastrointestinal regulation, antibacterial properties, anti-inflammatory effects, cytotoxicity, and antioxidant properties [3–6]. For nearly two centuries, agarwood has been extensively utilized as a treasured therapeutic perfume, in traditional medicine, as an aromatic food ingredient, and in religious incense across China, Japan, India, and other Southeast Asian countries [7]. Regrettably, within wild forests, the proportion of agarwood trees possessing resinous substances is merely 7% to 10%. Due to high demand, wild agarwood has been excessively harvested around the globe. All *Aquilaria. sinensis* (Lour.) Spreng (*A. sinensis*) and *Gyrinops* that can produce agarwood are at risk of extinction and are included in Appendix II of the Convention on Internal Trade in Endangered Species of Wild Fauna and Flora (CITES, http://checklist.cites.org (accessed on 12 July 2023)) [8]. Recently, a distinctive germplasm of *A. sinensis* has been identified from natural populations and employed as a scion for grafting in cultivation. These superior clones, known as Qi-Nan (alternatively referred to as Kanankoh, Kyara, and Chi-Nan), are esteemed as the finest quality of agarwood [9,10]. They are cherished for their enigmatic eastern fragrance, which is discernible even at room temperature without the need for combustion, unlike ordinary *A. sinensis* agarwood (OA) [11]. The high-grade Qi-Nan agarwood has substantially mitigated the shortage in the global supply.

Many studies focused on the differences between Qi-Nan and ordinary agarwood on germplasm genetics, incense smoke, and chemical composition [12–14], and have verified the high quality of Qi-Nan. The resin content of agarwood is a crucial quantitative criterion for grading the quality of agarwood [15]. Phenylethyl chromones with no or few substituents was one of the main factors contributing to agarwood fragrant, Qi-Nan agarwood contained 2-(2-phenylethyl) chromones with unsubstituted chromone rings [11,16]. The Qi-Nan agarwood exhibits comparable features, such as high contents of ethanol extracts and 2-(2-phenylethyl) chromone and 2-[2-(4-methoxyphenyl) ethyl] chromone, which were pivotal criteria for the high-quality agarwood [11]. Agarotetrol, a substituted PEC derivative, has demonstrated a positive correlation with agarwood quality and is employed as a biomarker for assessing agarwood [17,18]. According to the 2020 edition of the Pharmacopoeia of the People's Republic of China, agarotetrol content should meet the requirement of 0.1% [17]. The agarotetrol content was generally not exceeding 0.1% based on ten batches of Qi-Nan, whereas the ethanol extract content of Qi-Nan was much higher than the requirement of 10% in the pharmacopoeia, and the characteristics were consistent with the profiles of high-quality agarwood [19]. Inducing time was another important factor influencing agarwood quality, Yang et.al studied the characterization of 2-(2-Phenylethyl) chromone derivatives (PECs) for *Aquilaria crassna* agarwood, and verified 2-(2-phenylethyl) chromone and 2-[2-(4-methoxyphenyl) ethyl] chromone were upward as the inducing time extended [20]. Currently, a multitude of Qi-Nan clones are available in the market, each exhibited distinct aromas and quality attributes. Nevertheless, rare study investigated the differences in agarwood quality among various Qi-Nan agarwood clones, especially taking into account the inducing time.

Nowadays, artificial holing Qi-Nan agarwood from *A. sinensis* tree plays an extremely important role in the market [20]. This study detected and analyzed the concentration of ethanol extracts, agarotetrol, 2-(2-phenylethyl chromone) and 2-[2-(4-methoxy) phenylethyl] chromone contents in four grafted Qi-Nan agarwood clones with different inducing time after holing by using HPLC-MS/MS, and then revealed their agarwood quality. Our hypothesis was that the quality of Qi-Nan agarwood is influenced by both the specific clone and the inducing time, with key chemical markers such as ethanol extract content, agarotetrol, 2-(2-phenylethyl) chromone, and 2-[2-(4-methoxyphenyl) ethyl] chromone serving as reliable indicators of high-quality agarwood. This could be referenced for offering data support for agarwood quality evaluations and further elucidating the influence of various clones and inducing time on Qi-Nan quality, which may guide the selection of agarwood clones at varying inducing time and provide a fresh perspective for the differential study of natural products.

## 2. Materials and methods

### 2.1 Reagents and samples

The high-quality grafted Qi-Nan agarwood clones of YYZ, AS, RH, XGY used in this study were collected in Dianbai City, located in Guangdong Province, China (21°41′ N, 111°11′ E). These four types of grafted Qi-Nan agarwood clones are the most widely circulated in the market and also rank among the top in terms of cultivation area [22]. Nowadays, agarwood in trade mostly comes from cultivated *Aquilaria* trees induced by artificial methods, of which the traditional artificial holing is the most common and popular one [20]. In this study, healthy Qi-Nan trees, after growing for 3 years, were selected for agarwood production by drilling holes starting 20 cm above the ground with a diameter of 0.8–1.0 cm into the stems (S1 Fig in S1 File). The distance between each hole was 8.0–10.0 cm, and the drilling depth was two-thirds of the tree's diameter. Resin-enriched wood was meticulously harvested from the interior of chopped branches after induction for half a year, one year, and two years. Five trees were randomly sampled at various locations to ensure representativeness for each replicate. Three replicates were sampled for each inducing time and clone. YYZ-0.5, YYZ-1, and YYZ-2 represent the YYZ samples induced for 6, 12, and 24 months, respectively, with similar naming rules applied to AS, RH, and XGY clone. At the same time, we also collected OA samples with agarwood formation at 6 months and 10 months, as well as white wood agarwood (WWA) samples. Qi-Nan trees’ resinous wood from four grafted clones, OA and WWA were subjected to a High-Performance Liquid Chromatography with Tandem Mass Spectrometry (HPLC-MS/MS) analysis. All samples were pulverized into a powder and vacuum-dried overnight at room temperature. These powders were then sealed and stored at 4 °C until analysis. Standards of agarotetrol and 2-(2-phenyl) ethyl chromone were obtained from GlpBio (USA).

### 2.2 Ethanol extract content

To determine the ethanol extract content, 2 grams of the vacuum-dried agarwood powder were soaked in 100 mL of 95/5 (V/V) ethanol/water solution for 1 hour. The mixture was then connected to a reflux condenser, heated to boiling, and maintained at a gentle boil for 1 hour in one extraction cycle. The ethanol extract content was calculated as the dried weight of the extract divided by the dried weight of the powder (W/W %) [21]. The extract is also used for subsequent HPLC-MS/MS analysis.

### 2.3 HPLC-MS/MS analysis

#### 2.3.1 Preparation of standards solution.

The standard stock solutions of agarotetrol and 2-(2-phenylethyl) chromone were prepared in 95% ethanol. From the stock solutions, several working solutions were prepared by appropriate dilution in 95% ethanol. 2-(2-phenylethyl) chromone and 2-[2-(4-methoxyphenyl) ethyl] chromone share the same unsubstituted chromone skeleton [22,23]. The content of 2-[2-(4-methoxy) phenylethyl] chromone is calculated using the standard curve of 2-(2-phenylethyl) chromone [24–27]. All solutions were stored in a refrigerator at store at −20°C until analysis.

#### 2.3.2 HPLC-MS/MS method.

The analytical equipment utilized included an AB SCIEX API 4000 linear ion QTRAP quadrupole mass spectrometer (Foster City, CA, USA), operating in turbo electrospray ionization (ESI) mode. The chromatographic analyses were conducted using a Phenomenex C18 column (100 mm × 2.1 mm, 2.6 μm) alongside a trapping column at a flow rate of 0.5 mL/min. For the mobile phase, eluents consisting of 0.1% (V/V) formic acid (A) and acetonitrile (B) were utilized to enhance peak shape and separation efficiency. In Qi-Nan agarwood, the concentrations of agarotetrol and 2-(2-phenylethyl)chromone differ significantly; therefore, distinct chromatographic conditions were employed for their respective analyses. For agarotetrol, the optimized HPLC gradient elution program was as follows: 10% mobile phase B was maintained from 0 to 4 minutes, followed by a linear increase from 10% to 90% B between 4 and 5.9 minutes. The gradient then returned from 90% to 10% B over 5.9 to 6 minutes and was held at 10% B from 6 to 8 minutes for column re-equilibration. For the quantification of 2-(2-phenylethyl)chromone, the gradient started at 40% B from 0 to 1 minute, increased linearly to 60% B from 1 to 5 minutes, then returned to 40% B from 5 to 5.1 minutes, and was maintained at 40% B from 5.1 to 7 minutes for equilibration. The mass spectrometry parameters were set to the following: positive polarity scanning, desolvation temperature at 550°C, curtain gas flow at 25 μL/min, capillary voltage in negative ion mode at 5500V, spray gas at 55 psi, auxiliary heating gas also at 55 psi, interface heating switched on, and spray gas at a medium setting [28]. Detailed mass spectrometry parameters for the three components are presented in Table 2.

**Table 1:** Mass spectrometry parameters for agaroterol, 2-(2-phenylethyl) chromone and 2-[2-(4-methoxyphenyl) ethyl] chromone.

Compound	Molecular formula	m/z	Ionic mode	Collision energy (V)	Declustering potential (V)
Agaroterol	C_17_H_18_O_6_	319.1 > 91.1	ES+	63	60
2-(2-phenylethyl) chromone	C_17_H_14_O_2_	251.2 > 91.1	ES+	38	100
2-[2-(4-methoxyphenyl) ethyl] chromone	C_18_H_16_O_3_	281.2 > 121.1	ES+	38	120

**Table 2:** Result of validation procedure.

Analyte	Regression equation	R^2^ (n = 6)	Precision RSD (%) n = 6	Repeatability RSD (%) n = 6	Recovery (%) n = 6			Recovery RSD (%) n = 6		
					low	medium	high	low	medium	high
agarotetrol	y = 476.50 x + 1725.25	0.9998	1.45	2.43	100.05	100.00	99.54	3.10	1.61	1.14
2- (2-phenylethyl) chromone	y = 5.95e4x + 11751.95	0.9992	1.20	1.13	99.96	100.95	101.30	1.49	1.40	1.31

All data were analyzed using Analyst® 1.6.3, the software provided with the AB SCIEX HPLC/MS/MS system.

#### 2.3.3 Validation of the HPLC-MS/MS method.

The method was validated for linearity, precision, repeatability, and recovery [29]. The linearity evaluation was conducted in triplicate using standard solutions that corresponded to each point on the calibration curves. For agarotetrol and 2-(2-phenylethyl-chromone), the regression coefficients (R²) exceeded 0.9992 across five concentrations, ranging from 2 to 400 ng mL^-1^ (Table 1).

The precision and repeatability of the method, expressed as relative standard deviation (%RSD), were assessed through six replicates of a working standard solution (precision) and six identical sample extracts (repeatability) on the same day. The %RSD values for the working standard solution were 1.45% and 1.20%, while for the sample extracts, they were 2.43% and 1.13% for agarotetrol and 2-(2-phenylethyl) chromone respectively. These results demonstrated good precision and repeatability, falling within the acceptable variability limits for method analysis (Table 1). Recovery studies were conducted to assess the accuracy of the method. Samples of Qi-Nan agarwood were analyzed both before and after the addition of known amounts of agarotetrol and 2-(2-phenylethyl) chromone. The average recovery rates for agarotetrol in the low, medium, and high concentration ranges were 100.05%, 100.00%, and 99.54%, with %RSD values of 3.10%, 1.61%, and 1.14%, respectively. For 2-(2-phenylethyl) chromone, the average recovery rates in the low, medium, and high concentration ranges were 99.96%, 100.95%, and 101.30%, with %RSD of 1.49%, 1.40%, and 1.31%, respectively. These results demonstrate the good accuracy of the developed HPLC method (Table 1).

### 2.4 Statistical analysis

Statistical analysis was conducted using the SPSS software, version 24.0 (SPSS Software, SPSS Inc, Chicago, USA). The R platform (version 4.1.3) was used to perform the analysis of principal component analysis (PCA) and variance partitioning analysis (VPA).

In order to investigate the agarwood quality of different inducing time and clones, the Membership Function Values (MFVs) was used to comprehensively evaluate the agarwood quality through weighted analysis of components [30].

Weight was assigned to a particular indicator within the comprehensive context of evaluation. The process of calculating weight was [31]:


$$W_{i}=pc_{i}/\sum {\mathrm{\backslash nolimits}}_{i=1}^{n}pc_{i}$$

In the formula, *Wi* and *PCi* denote the weight and contribution rate, respectively, of the i-th comprehensive index within the set of all comprehensive indices; ‘*n*’ represents the count of principal components that have been extracted. The calculation of the Membership Function Value (MFV) in fuzzy mathematics is as follows:


$$\mu \left(X_{j}\right)=\left(X_{j}-X_{\min}\right)/\left(X_{\max}-X_{\min}\right)$$

*μ(Xj)* denotes the membership function value for the *j*-th composite index, with *Xj* being the value of the *j*-th composite index; *Xmin* and *Xmax* are the respective minimum and maximum values of the *j*-th composite index. The ultimate membership function value for the comprehensive assessment is given by:


$$MFV=\sum {\mathrm{\backslash nolimits}}_{j=1}^{n}\left[\mu \left(X_{j}\right)\times \left(W_{i}\right)\right]$$

## 3. Results

### 3.1 Morphological and genetic differentiation of Qi-Nan clones

**Fig 1:**
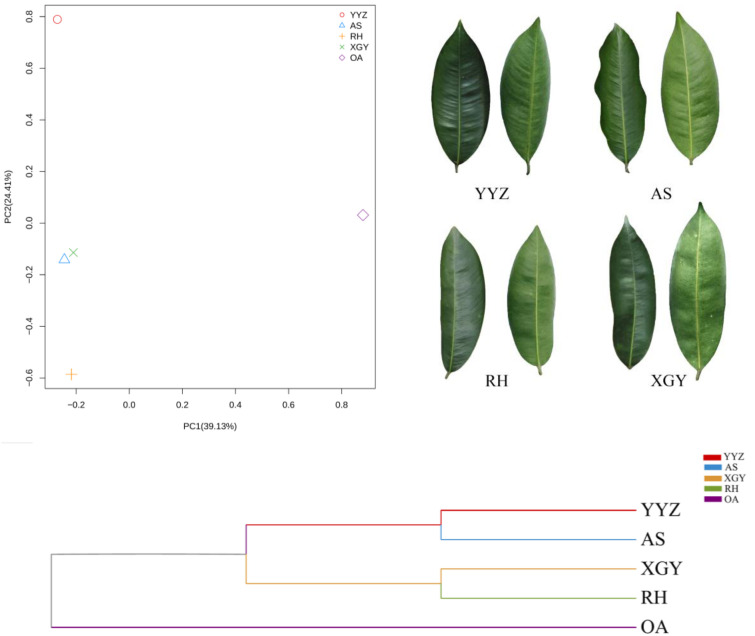
Principal component analysis (PCA) plot (top left) showing genetic differentiation among Qi-Nan clones and ordinary *A. sinensis* (OA). Morphological characteristics of the leaves of four Qi-Nan clones (top right); The phylogenetic treeexhibited the genetic relationships between OA and Qi-Nan clones, as well as the divergence among individual clones (bottom).

The morphological traits of these clones exhibited minimal variation. The leaves of YYZ are elliptical to oblong, featuring a symmetrical structure with a gradually tapered apex. In contrast, AS leaves display a spoon-shaped form with prominently undulating margins. RH leaves exhibit a subtle golden-yellow luster, while XGY leaves are characterized by a distinctive palmate-lobed morphology (Fig 1 ). Hu et al demonstrated the distinct growth characteristics of the four clones [32]. Through resequencing, PCA analysis revealed clear genetic differentiation among the Qi-Nan clones, with PC1 and PC2 accounting for 39.13% and 24.41% of the total genetic variance, respectively. The phylogenetic analysis further highlighted distinct differences between the OA and Qi-Nan clones, as well as genetic divergence among individual Qi-Nan clones. Overall, these four Qi-Nan clones exhibit genetic variation and can be clearly distinguished from OA at the genetic level (Fig 1).

### 3.2 Changes in ethanol extracts through inducing time and in response to Qi-Nan clones

**Fig 2:**
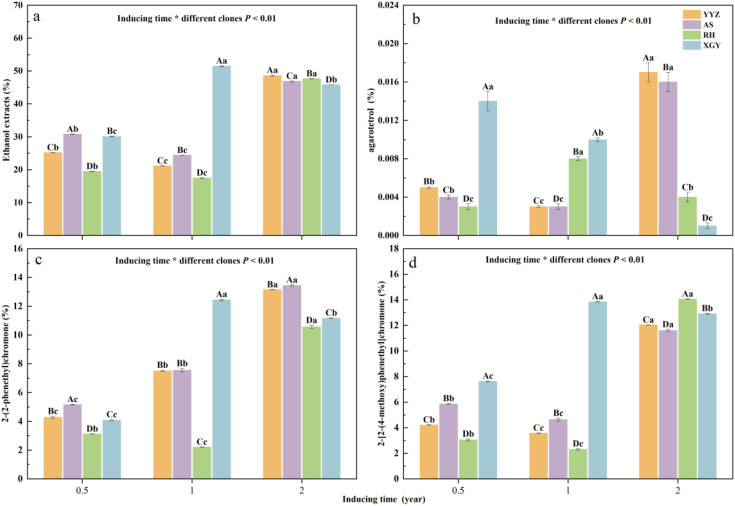
The content of ethanol extracts. **(a), agarotetrol (b),2-(2phenylethyl) chromone (c) and 2-[2-(4-methoxyphenyl) ethyl] chromone (d) across inducing time and different clone.** Bars represent standard error (n = 3). Analysis of variance (ANOVA) was conducted to test for statistically significant differences. The numbers used in the naming correspond to the inducing time, with 0.5 indicating half a year, 1 signifying one year, and 2 referring to two years. Capital letters above bars indicated differences among clones under same inducing time, Lowercase letters above bars represented differences among inducing time under same clone. Inducing time and different clone effects (two-way ANOVA) are indicated inside the panels.

Ethanol extracts content varied among different Qi-Nan agarwood clones and inducing time. For YYZ, AS, and RH, the ethanol extract content significantly declined from half a year to one year, and reached its highest level after two years of induction, with levels exceeding 46%. XGY showed different trends, with a significant increase in ethanol extract content from half a year to one year of induction, followed by a notable decrease from one year to two years. The ethanol extract content for XGY reached its peak at 51.49% after one year of induction. Apart from the XGY clone, YYZ, AS, and RH followed a consistent pattern of changes in extract content, with AS>YYZ > RH after half a year and one year of induction. After two years of induction, YYZ exhibited the greatest ethanol extract content, showing enhancements of 1.77%, 0.91%, and 2.75% over AS, RH, and XGY, respectively (Fig 2A ). The ethanol extract content of OA after half a year of agarwood formation was 18.16%, which is lower than that of all Qi-Nan clones. The ethanol extract content of WWA was 3.39% (S2a Fig in S1 File).

### 3.3 Variation in agarotetrol content across inducing time and Qi-Nan clones

Compared with Qi-Nan agarwood, the content of agarotetrol in OA, whether formed for half a year or 10 months, exceeded 0.1%, which is higher than that of Qi-Nan agarwood at all formation stages in this study. Agarotetrol were not detected in WWA (S2b Fig in S1 File). The Qi-Nan clones and inducing time both had significant effects on the concentration of agarotetrol. After half a year of induction, the concentration of agarotetrol in XGY was significantly higher than the others. With the exception of RH, all samples showed a significant decrease in concentration after one year of induction. After two years of induction, YYZ had the highest concentration of agarotetrol, while XGY had the lowest. From half a year to two years of induction, YYZ and AS exhibited a trend of initially decreasing and then increasing, with the concentration after two years being 2.30 and 1.92 times than that of one year, respectively. RH showed a trend of first increasing and then decreasing, whereas XGY demonstrated a gradual decline in concentration, with the concentration after two years being only 8.72% of that after half a year (Fig 2b).

### 3.4 Changes in the concentrations of the two PECs

The content of 2-(2-phenethyl) chromone and 2-[2-(4-methoxy) phenethyl] chromone are two crucial PECs for evaluating the quality of Qi-Nan. From the diagram, it was apparent that as the inducing time increased, the concentrations of 2-(2-phenethyl) chromone and 2-[2-(4-methoxy) phenethyl] chromone exhibited varying trends of change, depending on the particular Qi-Nan clone (Fig 2c, 2d). For the concentration changes of 2-(2-phenethyl) chromone, both YYZ and AS showed a consistent uptrend, while RH displayed a pattern of initially decreasing and then increasing, unlike other clones, XGY reached its peak at one year of induction and then decreased again after two years of induction. As for the change in concentration of 2-[2-(4-methoxy) phenethyl] chromone, YYZ, AS, and RH showed the same trend, decreased from half a year to one year of induction and reached a peak after two years of induction. XGY, however, reached to its peak at one year, which was consistent with the pattern observed for 2-(2-phenethyl) chromone concentration. The concentrations of XGY 2-(2-phenethyl) chromone and 2-[2-(4-methoxy) phenethyl] chromone after one year of induction were only 1.00% and 0.21% lower than AS and RH after two years of induction respectively (Fig 2c, 2d). At all agarwood formation stages, the content of the two PECs in Qi-Nan agarwood was more than ten times higher than that in OA. For OA, the content of 2-[2-(4-methoxyphenyl) ethyl] chromone gradually increased from half a year to 10 months of formation. These two PECs were not detected in WWA (S2c, S2d Fig in S1 File).

### 3.5 Evaluation of Qi-nan agarwood quality of different inducing time and clones

**Fig 3:**
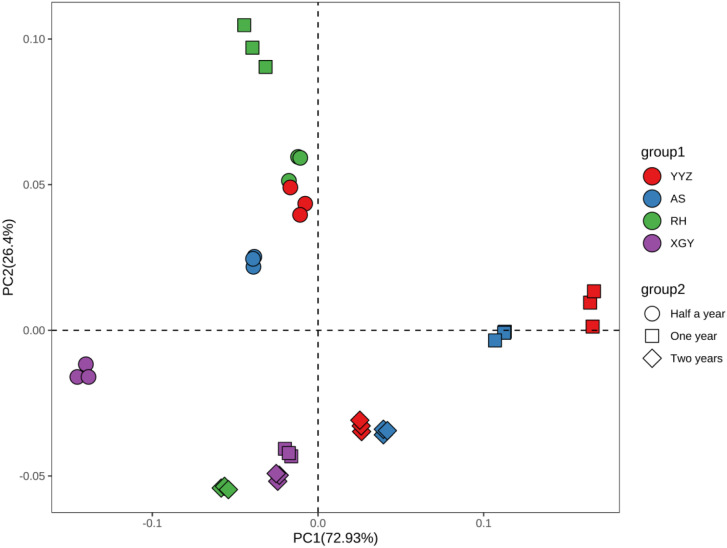
Principle component analysis (PCA) for the different clone of Qi-Nan and inducing time, group 1 represents Qi-Nan clones, and group 2 represents inducing time.

**Fig 4:**
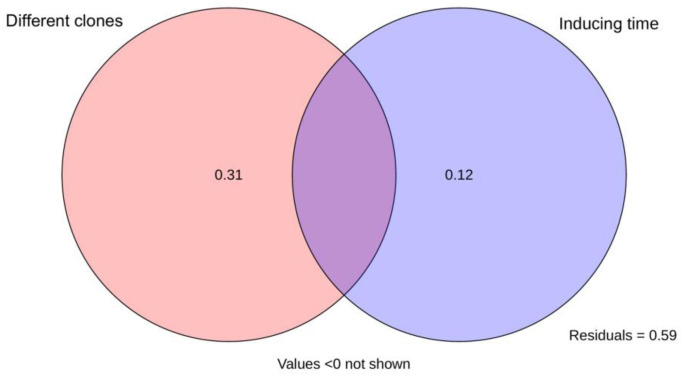
Agarwood quality of Qi-Nan by membership function value (MFV). The 12 treatments are labeled according to a clone-inducing time scheme, with the four clones being YYZ, AS, RH, and XGY, where 0.5 denotes half a year, 1 denotes one year, and 2 denotes two years.

**Fig 5:**
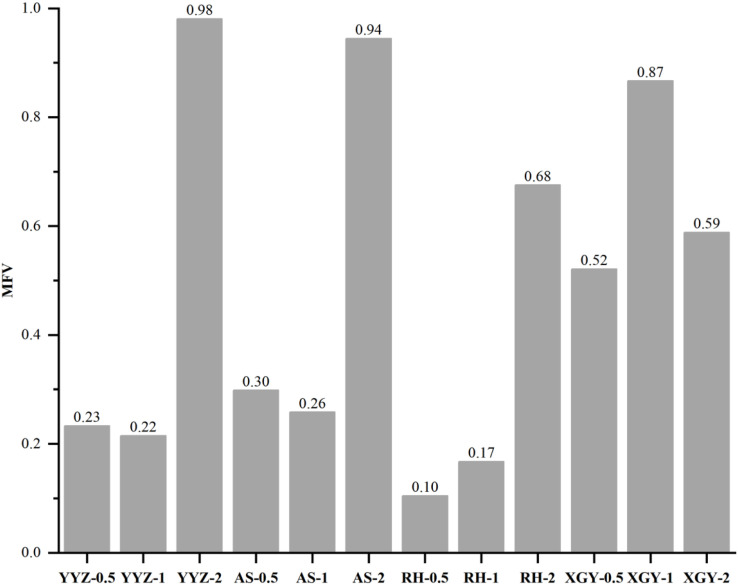
A variance partitioning analysis (VPA), illustrating effect of different clones, and inducing time factors on Qi-Nan agarwood quality.

PCA analysis was conducted based on the ethanol extract content, agarotetrol content, 2-(2-phenylethyl) chromone content and 2-[2-(4-methoxy) phenylethyl] chromone content, which showed that YYZ, AS and RH clustered together apart from XGY after half a year of induction, there was a great difference between clones after one year of induction, XGY-1 clustered with all clones after two years of induction (Fig 3). The MFV analysis was also used to evaluate agarwood quality of Qi-Nan at different inducing time and clone in the present study. A higher MFV indicates a better agarwood quality (Fig 4). The results showed that YYZ-2 had the highest MFV followed by AS-2, XGY-1, RH-2 and XGY-2, except for XGY, the agarwood quality reached the highest quality after two years of induction, XGY-1 agarwood quality was just lower 0.11 of MFV than YYZ-2. The quality of agarwood was affected by both the inducing time and the clone, which the difference of clone accounted for 31% and the inducing time was 12% (Fig 5).

## 4. Discussion

### 4.1 Agarwood quality varied among different Qi-Nan clones

The quality of agarwood has traditionally been assessed using subjective grading indicators such as tactile properties, water submersion behavior, color, fragrance, and shape [33]. However, these indicators might not be sufficient for accurate quality assessment of Qi-Nan, and there is an urgent need for more objective parameters, including chemical constituents. Recent advances have focused on metabolite-based quality control of agarwood, as research indicated a strong link between agarwood quality and its metabolite composition [34,35]. This study investigated the impact of different Qi-Nan clones on the quality of agarwood after half a year, one year, and two years of resin formation. The results confirmed our hypothesis: there are distinct differences in the quality of agarwood between different Qi-Nan clones at each inducing time. These differences were validated the differences in their quality through ethanol extracts, agarotetrol, 2-(2-phenethyl) chromone and 2-[2-(4-methoxy) phenethyl] chromone content. The resin content and composition within Qi-Nan are critical for determining the quality as well as establishing its market price [36]. The occurrence of agarotetrol can signify whether an agarwood plant has accumulated resin, as it is not found in healthy agarwood plants and is presumably produced during cellular demise. Of the two main constituents in Qi-Nan, 2-(2-phenylethyl) chromone and 2-[2-(4-methoxyphenyl) ethyl] chromone, which constitute the largest proportion, these compounds emit a fragrant aroma upon heating and are fundamental to the grading of agarwood quality [37–39]. Agarwood is a secondary metabolite produced as a result of the defense response in *Aquilaria* species. Its formation involves a trade-off between growth and defense mechanisms. Growth processes supply the necessary carbon skeletons for agarwood production, while the defense response transforms these precursors into the secondary metabolites that determine the quality of the agarwood [40]. The XGY clone exhibited the best agarwood quality after half a year and one year of induction among the four clones, while after two years of induction, the YYZ clone had the best agarwood quality (Fig 5). Our analysis revealed clear genetic differentiation was revealed among the four Qi-Nan clones through resequencing (Fig 1), The observed differences in agarwood quality are likely due to genetic variations that affect key anatomical features such as the size and efficiency of interxylary phloem and xylem rays, these anatomical traits play a critical role in the accumulation of agarwood resin [41]. Many studies have compared various Qi-Nan clones with ordinary agarwood to assess their component profiles and overall quality [42,43]. However, our findings indicate significant variability in quality even among different Qi-Nan clones, underscoring the importance of clone-specific factors in determining resin quality. Genetic differences between clones may influence their resin synthesis capacities, which helps explain the distinct quality of agarwood produced at varying inducing times. The stress response, which governs resin accumulation, is a complex process involving various factors such as signaling pathways, transcription factors, hormones, enzymes, and secondary metabolites [44]. Future studies should aim to further elucidate these underlying mechanisms to better understand how these factors contribute to the quality variability observed among Qi-Nan clones.

### 4.2 The impact of different clones and inducing time on the quality of Qi-Nan agarwood

This research examined how the inducing time and the various clones of the Qi-Nan affected agarwood quality. Two-way analysis of variance indicated that different clones and inducing time both had significant effects on their contents (Fig 2). After drilling, the tree body of the agarwood is damaged, and some endophytic fungi grow rapidly, triggering the defensive response of the agarwood tree [45]. The starch granules in the active cells of the xylem are converted into non-starch polysaccharides and phenolics. These intermediates undergo a series of chemical reactions, transforming into chromones and sesquiterpenes [46]. During this process, jasmonic acid (JA), produced by the endophytic strains in *A. sinensis*, acts as a crucial signaling molecule that initiates the biosynthesis of sesquiterpenes and chromone derivatives, thereby affecting the chemical composition and quality of agarwood [47,48]. Studies have shown that the endophytic fungi in Qi-Nan agarwood are significantly positively correlated with the yield, 2-(2-phenylethyl) chromone content, with the dominant fungal communities being *Devriesia* at the onset of agarwood formation, followed by *Devriesia*, *Arthopyrenia*, and *Acremonium* after 12 months, and *Devriesia*, *Pseudoteratosphaeria*, and *Morenoina* after 24 months of agarwood formation [49]. The results showed that the content of ethenal extracts, agarotetrol and chromone content did not increase with longer inducing time, indicating that there was no positive correlation between inducing time and Qi-Nan quality. The differences in agarwood quality observed in this study are likely related to the variations in endophytic fungi at different agarwood formation times. The variance partitioning analysis (VPA) result indicated that inducing time accounted for 12% of the impact on Qi-Nan quality (Fig 4). Except for the XGY clone, the other three clones clustered together after half a year of induction, with the XGY clone containing higher levels of ethanol extract and 2-[2-(4-methoxyphenyl) ethyl] chromone (Fig. 3). From half a year to one year, most Qi-Nan clones showed a decrease in quality. After one year of induction, there was significant differentiation among the four clones, with the ethanol extract content of XGY reaching to 51.49%, which was close to the ethanol extract content in agarwood of wild *Aquilaria* tree [50,51], and higher than all the two-year induced Qi-Nan clones. 2-(2-phenylethyl) chromone and 2-[2-(4-methoxyphenyl) ethyl] chromone content were up-regulated as inducing time increased for YYZ and AS clones, it was consistent with previous study that the relative contents of flindersia type 2-(2-phenylethyl)chromones (FTPECs), especially for these two FTPECs mentioned above, were rised for the *Aquilaria crassna* samples from two, four and five years of the agarwood formation times [20], However, this trend did not occur in XGY and RH. During the activation of the defense response, defensive compounds such as chromones reduce the abundance of fungi, thereby weakening the defense capacity of A. sinensis, accompanied by a decline in the synthesis rate of chromones [52]. This may constitute the primary reason for the reduction in chromone content after prolonged induction for XGY and RH. Furthermore, chromones can undergo higher degrees of hydroxylation or methoxylation to form other types of chromones, and this transformation process may also contribute to the decrease in the content of the 2-(2-phenylethyl) chromone and 2-[2-(4-methoxyphenyl) ethyl] chromone [53]. From the VPA analysis, the difference of clone accounted for 31% of the influence on Qi-Nan quality, which was more than twice the influence of inducing time. (Fig 4). Two years of induction for YYZ, AS, and RH, and one year of induction for XGY clustered together (Fig 3), suggesting that the XGY clone can achieved higher quality more rapidly and can be harvested earlier. For other clones, a longer inducing time was needed. Further investigation into fungal community changes, jasmonic acid metabolic pathways will be necessary to explain why XGY reaches better agarwood quality faster. This provided some scientific references for the industrial development of Qi-Nan agarwood.

## 5. Conclusions

Qi-Nan has emerged in the agarwood market as a newly recognized high-quality type of agarwood. In this study, we used HPLC-MS/MS to compare the chemical constituents of four Qi-Nan clones collected from Dianbai, Guangdong Province, which revealed several significant differences between the Qi-Nan clone and the inducing time for agarwood formation. To sum up, Qi-Nan exhibited consistent qualities, notably the high concentrations of ethanol extracts and two PECs of (2-(2-phenylethyl) chromone and 2-[2-(4-methoxyphenyl) ethyl] chromone), which are essential indicators of high-quality agarwood. At the same time, the content of agarotetrol was found to be low, lower than that of ordinary *A. sinensis*. The results showed several noteworthy differences in chemical constituents between the four Qi-Nan clones and different inducing time. The inducing time had a 12% impact while the difference of clone had a 31% impact on the quality of Qi-Nan. In this study, we selected three different inducing time of half a year, one year, and two years. For the majority of clones (YYZ, AS and RH), the quality of agarwood was unstable at half a year and one year, the quality of agarwood of these clones were the best after two years; however, for the XGY clone, the quality was the best after one year of induction. It indicated that XGY can achieve high quality in a shorter inducing time. These results are helpful for evaluating the impact of different clones and inducing time on the quality of Qi-Nan collected in Dianbai, Guangdong province, and for the advancement of the agarwood industry.

## Supporting information

S1 File Material and method for resequencing. (RAR)

## References

[1] De Alwis WNH. Variation of naturally and artificially induced agarwood resin content and quality of *Gyrinops walla* for commercial extraction and its seed germination. 2017.

[2] Okugawa H, Ueda R, Matsumoto K, Kawanishi K, Kato A. Effects of agarwood extracts on the central nervous system in mice. Planta Med. 1993;59(1):32–6. doi: 10.1055/s-2006-959599 8441779

[3] Lin Z, Li H, Mei Q. Comparative study on antiinflammatory of agarwood leaves and resin. Chin Arch Tradit Chin Med. 2013;31:548–9.

[4] Dahham SS, Hassan LEA, Ahamed MBK, Majid ASA, Majid AMSA, Zulkepli NN. In vivo toxicity and antitumor activity of essential oils extract from agarwood (*Aquilaria crassna*). BMC Complement Altern Med. 2016;16:236. doi: 10.1186/s12906-016-1210-1 27450078 PMC4957886

[5] Dahham SS, Tabana YM, Iqbal MA, Ahamed MBK, Ezzat MO, Majid ASA, et al. The anticancer, antioxidant and antimicrobial properties of the sesquiterpene β-Caryophyllene from the essential oil of *Aquilaria crassna*. Molecules. 2015;20(7):11808–29. doi: 10.3390/molecules200711808 26132906 PMC6331975

[6] Li W, Cai C-H, Dong W-H, Guo Z-K, Wang H, Mei W-L, et al. 2-(2-Phenylethyl)chromone derivatives from Chinese agarwood induced by artificial holing. Fitoterapia. 2014;98:117–23. doi: 10.1016/j.fitote.2014.07.011 25068202

[7] Hashim YZH-Y, Kerr PG, Abbas P, Mohd Salleh H. *Aquilaria* spp. (agarwood) as source of health beneficial compounds: a review of traditional use, phytochemistry and pharmacology. J Ethnopharmacol. 2016;189:331–60. doi: 10.1016/j.jep.2016.06.055 27343768

[8] Liu J, Yang J, Jiang C, Zhou J, Zhao Y, Huang L. Volatile organic compound and endogenous phytohormone characteristics during callus browning in *Aquilaria sinensis*. Ind Crop Prod. 2021;168:113605. doi: 10.1016/j.indcrop.2021.113605

[9] Yamagata E, Yoneda K. Pharmacognostical studies on the crude drug of agarwood (VI): On “Kanankoh”. Shoyakugaku Zasshi. 1987;41(2):142–6.

[10] Barden A, Anak NA, Mulliken T, Song M. Heart of the matter: agarwood use and trade and CITES implementation for *Aquilaria malaccensis*. Cambridge, UK: Traffic International; 2000.

[11] Mei WL, Zeng YB, Guo ZK, Zhao YX, Wang H, Zuo WJ, et al. 2-(2-phenylethyl) chromone derivatives in Chinese agarwood “Qi-Nan” from *Aquilaria sinensis*. Planta Med. 2013;79(14):1329–34. doi: 10.1055/s-0033-1351256 23929247

[12] Yang J, Dong W, Chen H, Kong F, Wang J, Mei W, et al. Qualitative and quantitative analysis of flidersiachromones in three agarwood samples by HPLC-MS/MS. Chem Res Chin Univ. 2018;34(3):389–96. doi: 10.1007/s40242-018-7273-4

[13] Chen F, Huang Y, Luo L, Wang Q, Huang N, Zhang Z, et al. Comprehensive comparisons between grafted kynam agarwood and normal agarwood on traits, composition, and in vitro activation of AMPK. Molecules. 2023;28(4):1667. doi: 10.3390/molecules28041667 36838655 PMC9961698

[14] Yu M, Liu Y, Feng J, Chen D, Yang Y, Liu P, et al. Remarkable phytochemical characteristics of Chi-Nan agarwood induced from new-found chi-nan germplasm of *Aquilaria sinensis* compared with ordinary agarwood. Int J Anal Chem. 2021;2021:5593730. doi: 10.1155/2021/5593730 33927765 PMC8053051

[15] Azah MN, Husni SS, Mailina J, Sahrim L, Majid JA, Faridz ZM. Classification of agarwood (gaharu) by resin content. J Trop For Sci. 2013;:213–9. doi: 10.2307/23617036

[16] Chen Y, Yan T, Zhang Y, Wang Q, Li G. Characterization of the incense ingredients of cultivated grafting Kynam by TG-FTIR and HS-GC-MS. Fitoterapia. 2020;142:104493. doi: 10.1016/j.fitote.2020.104493 32045691

[17] Commission CP. Chinese pharmacopoeia. 2020. Beijing, China: China Medical Science and Technology Press; 2020. 192–3.

[18] Takamatsu S, Ito M. Agarotetrol in agarwood: its use in evaluation of agarwood quality. J Nat Med. 2020;74(1):98–105. doi: 10.1007/s11418-019-01349-w 31392566

[19] Feng J, Hou W, Chen L, Chen X, Yang Y, Liu Y. Quality analysis and evaluation of agarwood produced by “Chi-nan” germplasm based on standard of Chinese Pharmacopoeia. Mod Chin Med. 2022;24:432–7. doi: 10.13313/j.issn.1673-4890.20210703002

[20] Yang J, Dong W, Kong F, Liao G, Wang J, Li W, et al. Characterization and analysis of 2-(2-Phenylethyl)-chromone derivatives from Agarwood (*Aquilaria crassna*) by artificial holing for different times. Molecules. 2016;21(7):911. doi: 10.3390/molecules21070911 27420040 PMC6273224

[21] Nguyen TTN, Trinh NY, Le PK. Recovery yield and bioactivities evaluation on essential oil and ethanolic extract of star anise (Illicium verum Hook. f.). CET J Chem Eng Trans. 2021;83. doi: 10.3303/CET2183025

[22] Naef R. The volatile and semi‐volatile constituents of agarwood, the infected heartwood of *Aquilaria* species: a review. Flavour Fragrance J. 2011;26(2):73–87. doi: 10.1002/ffj.2034

[23] Chen H-Q, Wei J-H, Yang J-S, Zhang Z, Yang Y, Gao Z-H, et al. Chemical constituents of agarwood originating from the endemic genus *Aquilaria* plants. Chem Biodivers. 2012;9(2):236–50. doi: 10.1002/cbdv.201100077 22344902

[24] Gao Y, Liu J, Lu H, Wei Z. Two New 2‐(2‐Phenylethyl)chromen‐4‐ones from *Aquilaria sinensis* (Lour.) Gilg. Helvetica Chimica Acta. 2012;95(6):951–4. doi: 10.1002/hlca.201100442

[25] Chen D, Xu Z, Chai X, Zeng K, Jia Y, Bi D, et al. Nine 2‐(2‐Phenylethyl)chromone derivatives from the resinous wood of *Aquilaria sinensis* and their inhibition of LPS‐induced NO production in RAW 264.7 cells. Eur J Org Chem. 2012;2012(27):5389–97. doi: 10.1002/ejoc.201200725

[26] Yoon JS, Lee MK, Sung SH, Kim YC. Neuroprotective 2-(2-phenylethyl)chromones of *Imperata cylindrica*. J Nat Prod. 2006;69(2):290–1. doi: 10.1021/np0503808 16499335

[27] Wang T, Li LF, Zhang K, Zhang WY, Pei YH. New 2-(2-phenylethyl) chromones from *Bothriochloa ischaemum*. J Asian Nat Prod Res. 2001;3(2):145–9. doi: 10.1080/10286020108041382 11407814

[28] Liu J, Yang J, Jiang C, Zhou J, Zhao Y, Huang L. Volatile organic compound and endogenous phytohormone characteristics during callus browning in *Aquilaria sinensis*. Indust Crops Prod. 2021;168:113605. doi: 10.1016/j.indcrop.2021.113605

[29] Peris‐Vicente J, Esteve‐Romero J, Carda‐Broch S. Validation of analytical methods based on chromatographic techniques: an overview. In: Analytical separation science. Wiley; 2015. 1757–808. doi: 10.1002/9783527678129.assep064

[30] Li X, Ullah S, Chen N, Tong X, Yang N, Liu J, et al. Phytotoxicity assessment of dandelion exposed to microplastics using membership function value and integrated biological response index. Environ Pollut. 2023;333:121933. doi: 10.1016/j.envpol.2023.121933 37277069

[31] Zhang X, Wang J, Feng S, Yu X, Zhou A. Morphological and physiological responses of *Dianthus spiculifolius* high wax mutant to low-temperature stress. J Plant Physiol. 2022;275:153762. doi: 10.1016/j.jplph.2022.153762 35820348

[32] Li X, Cui Z, Liu X, Hong Z, Zhang P, Xu D. Comparative morphological, anatomical and physiological analyses explain the difference of wounding-induced agarwood formation between ordinary agarwood nongrafted plants and five grafted Qi-Nan clones (*Aquilaria sinensis*). Forests. 2022;13(10):1618. doi: 10.3390/f13101618

[33] Xie ZW. Description of varieties of chinese medicinal materials. Shanghai Sci Technol Press; 1964.

[34] Azren PD, Lee SY, Emang D, Mohamed R. History and perspectives of induction technology for agarwood production from cultivated *Aquilaria* in Asia: a review. J For Res. 2018;30(1):1–11. doi: 10.1007/s11676-018-0627-4

[35] Wang S, Yu Z, Wang C, Wu C, Guo P, Wei J. Chemical constituents and pharmacological activity of agarwood and *Aquilaria* plants. Molecules. 2018;23(2):342. doi: 10.3390/molecules23020342 29414842 PMC6017114

[36] Wang Y, Hussain M, Jiang Z, Wang Z, Gao J, Ye F, et al. *Aquilaria* Species (Thymelaeaceae) distribution, volatile and non-volatile phytochemicals, pharmacological uses, agarwood grading system, and induction methods. Molecules. 2021;26(24):7708. doi: 10.3390/molecules26247708 34946790 PMC8703820

[37] Batubara R, Hanum TI, Affandi O. GC-MS analysis of young and mature wild agarwood leaves (*Aquilaria malaccensis* Lamk) and its antioxidant potential. IOP Conf Ser: Earth Environ Sci. 2021;912(1):012038. doi: 10.1088/1755-1315/912/1/012038

[38] Li W, Chen H-Q, Wang H, Mei W-L, Dai H-F. Natural products in agarwood and *Aquilaria* plants: chemistry, biological activities and biosynthesis. Nat Prod Rep. 2021;38(3):528–65. doi: 10.1039/d0np00042f 32990292

[39] Ishihara M, Tsuneya T, Uneyama K. Components of the volatile concentrate of agarwood. J Essential Oil Res. 1993;5(3):283–9. doi: 10.1080/10412905.1993.9698221

[40] Zhang P, Li X, Cui Z, Xu D. Morphological, physiological, biochemical and molecular analyses reveal wounding-induced agarwood formation mechanism in two types of *Aquilaria sinensis* (Lour.) spreng. Indust Crops Prod. 2022;178:114603. doi: 10.1016/j.indcrop.2022.114603

[41] Rasool S, Mohamed R. Understanding agarwood formation and its challenges. In: Tropical forestry. Springer Singapore; 2016. 39–56. doi: 10.1007/978-981-10-0833-7_3

[42] Li X, Cui Z, Liu X, Hong Z, Zhang P, Xu D. Comparative morphological, anatomical and physiological analyses explain the difference of wounding-induced agarwood formation between ordinary agarwood nongrafted plants and five grafted Qi-Nan clones (*Aquilaria sinensis*). Forests. 2022;13(10):1618. doi: 10.3390/f13101618

[43] Lv F, Yang Y, Sun P, Zhang Y, Liu P, Fan X, et al. Comparative transcriptome analysis reveals different defence responses during the early stage of wounding stress in Chi-Nan germplasm and ordinary *Aquilaria sinensis*. BMC Plant Biol. 2022;22(1):464. doi: 10.1186/s12870-022-03821-4 36171555 PMC9520901

[44] Cui J, Li X, Lu Z, Jin B. Plant secondary metabolites involved in the stress tolerance of long-lived trees. Tree Physiol. 2024;44(2):tpae002. doi: 10.1093/treephys/tpae002 38196002

[45] Chhipa H, Deshmukh SK. Diversity of endophytic fungi and their role in artificial agarwood production in *Aquilaria* tree. In: Advancing frontiers in mycology and mycotechnology. Springer Singapore; 2019. 479–94. doi: 10.1007/978-981-13-9349-5_19

[46] Ma S, Fu Y, Li Y, Wei P, Liu Z. The formation and quality evaluation of agarwood induced by the fungi in *Aquilaria sinensis*. Indust Crops Prod. 2021;173:114129. doi: 10.1016/j.indcrop.2021.114129

[47] Tan CS, Isa NM, Ismail I, Zainal Z. Agarwood induction: current developments and future perspectives. Front Plant Sci. 2019;10:122. doi: 10.3389/fpls.2019.00122 30792732 PMC6374618

[48] Zhang X, Wang LX, Hao R, Huang JJ, Zargar M, Chen M-X, et al. Sesquiterpenoids in agarwood: biosynthesis, microbial induction, and pharmacological activities. J Agric Food Chem. 2024;72(42):23039–52. doi: 10.1021/acs.jafc.4c06383 39378105

[49] Li X, Fang X, Cui Z, Hong Z, Liu X, Li G, et al. Anatomical, chemical and endophytic fungal diversity of a Qi-Nan clone of *Aquilaria sinensis* (Lour.) Spreng with different induction times. Front Plant Sci. 2024;15:1320226. doi: 10.3389/fpls.2024.1320226 38590741 PMC10999641

[50] Liu Y, Chen H, Yang Y, Zhang Z, Wei J, Meng H, et al. Whole-tree agarwood-inducing technique: an efficient novel technique for producing high-quality agarwood in cultivated *Aquilaria sinensis* trees. Molecules. 2013;18(3):3086–106. doi: 10.3390/molecules18033086 23470337 PMC6270329

[51] Liu Y, Yang Y, Wei J, Zhang Z, Chen B. Analysis on the quality of agarwood produced via the whole-tree agarwood-inducing technique in different areas. Mod Chin Med. 2014;16:183–6. doi: 10.13313/j.issn.1673-4890.2014.03.002

[52] Morris H, Brodersen C, Schwarze FWMR, Jansen S. The parenchyma of secondary xylem and its critical role in tree defense against fungal decay in relation to the CODIT model. Front Plant Sci. 2016;7:1665. doi: 10.3389/fpls.2016.01665 27881986 PMC5101214

[53] Oishi S, Kodera Y, Nishikawa H, Kamitani H, Watabe T, Ohno H, et al. Design and synthesis of membrane fusion inhibitors against the feline immunodeficiency virus. Bioorg Med Chem. 2009;17(14):4916–20. doi: 10.1016/j.bmc.2009.06.001 19541488

